# Plasticity of Hippocampal Excitatory-Inhibitory Balance: Missing the Synaptic Control in the Epileptic Brain

**DOI:** 10.1155/2016/8607038

**Published:** 2016-02-24

**Authors:** Christian Bonansco, Marco Fuenzalida

**Affiliations:** ^1^Centro de Neurobiología y Plasticidad Cerebral (CNPC), Instituto de Fisiología, Facultad de Ciencias, Universidad de Valparaíso, Gran Bretaña Avenida 1111, 2360102 Valparaíso, Chile; ^2^Núcleo Milenio en Biología de Enfermedades Neurosiquiátricas, Instituto de Fisiología, Facultad de Ciencias, Universidad de Valparaíso, Gran Bretaña Avenida 1111, 2360102 Valparaíso, Chile

## Abstract

Synaptic plasticity is the capacity generated by experience to modify the neural function and, thereby, adapt our behaviour. Long-term plasticity of glutamatergic and GABAergic transmission occurs in a concerted manner, finely adjusting the excitatory-inhibitory (E/I) balance. Imbalances of E/I function are related to several neurological diseases including epilepsy. Several evidences have demonstrated that astrocytes are able to control the synaptic plasticity, with astrocytes being active partners in synaptic physiology and E/I balance. Here, we revise molecular evidences showing the epileptic stage as an abnormal form of long-term brain plasticity and propose the possible participation of astrocytes to the abnormal increase of glutamatergic and decrease of GABAergic neurotransmission in epileptic networks.

## 1. Introduction

Epilepsy is characterized by spontaneous recurrent seizures and comprises a diverse group of syndromes with different aetiologies [1]. Epilepsy is the second most common brain disorder, affecting about 1% of the world's population [2]. Temporal lobe epilepsy (TLE) remains as one of the most severe and frequent pharmacoresistant types of focal acquired epilepsies. The recurrent seizure is an electrographic hallmark of several types of epilepsy, which consist in an excessive synchronous discharge of cerebral neurons, generated in one or more neuron populations (i.e., epileptic focus) [3]. The electrical activity in epileptic network is associated with an E/I synaptic imbalance, which promotes neuronal hyperexcitability and hypersynchronization, through an increase in excitatory neurotransmission as well as decrease of inhibitory neurotransmission and/or GABA-mediated hyperexcitability [4–6]. During the seizures, associated with heightened neuronal excitability and abnormal synchronization of discharge in the epileptic focus, the disruption of brain functions occurs [7]. Much of the knowledge about neurobiology of epilepsy has been attained from resected temporal lobe tissues from patients, whereas the cellular basis of epilepsy has been obtained from acute experimental models of seizures (i.e., ictogenesis) [8, 9], which contrasts with the limited understanding of neurobiological mechanisms of epilepsy development (epileptogenesis) [10, 11] (see Box  1). Both inhibitory and excitatory synapses are found to exhibit important changes that can mediate the initiation and evolution of self-sustaining seizures. The synaptic plasticity is essential to normal brain function such as our ability to learn and modify our behaviour. Several evidences have showed that astrocytes can modulate the synaptic plasticity and excitability in both excitatory and inhibitory synaptic circuits [12–14]. Currently, experimental evidence suggests that the start, progress, and consolidation of epileptic stage could overlap with the mechanisms underlying the long-term plasticity, learning, and memory [15, 16], which could be explained by an alteration of the factors that regulate the synaptic plasticity of excitatory and inhibitory circuits. Here, we will review the main evidences in those cellular and molecular alterations with focus on the synaptic plasticity that conducts to E/I imbalance and a pronounced vulnerability of the brain to epilepsy.

*Box  1*

*Kindling and Long-Term Plasticity in Hippocampal Formation.* Epilepsy encloses a set of neurological disorders of diverse aetiology, characterized by the development of gradual and progressive spontaneous seizures, which increase in recurrence and severity with time. To study epilepsy, both acute and chronic models have been developed [17]. Kindling, one of the chronic models of experimental epileptogenesis more extensively used, can be induced either* in vivo* (i.e., freely moving rats) [8, 18–20] or* in vitro* (i.e., brain slices) [21–23], allowing reproducing the progressive development of disease. The kindling protocol consists in the repetitive presentation of stimuli (either chemical or electrical) on a nervous structure, usually amygdala or hippocampus, at subconvulsive intensities, which elicits gradual and progressive augmentation of electroencephalographic (EEG) activity after stimuli and behavioural. In several pharmacological and acute models of epileptogenesis, the epileptic state is reached after spontaneous recurrent seizures [24, 25]. EEG activity corresponding to tonic-clonic firing of population spikes, namely, after discharge (AD), can be detected in stimulated structure as well as in projection areas. This AD or electrographic seizure (i.e., EGSs* in vitro*) has been attributed to ictal discharges as product to the increase of synchronous activity and hyperexcitability of a large group of neurons [26]. The long-term changes in the synaptic efficacy are activity-dependent of network and can produce either facilitation or depression, depending on the stimulus parameters and repetition [27]. In hippocampal neurons, long-term potentiation (LTP) of glutamatergic synapses produces the strengthening of synaptic efficacy, which can be induced by high-frequency stimulation or by coincidence between pre- and postsynaptic activity. As well as in excitatory synapses, the neuronal activity can trigger LTP or LTD of GABAergic synaptic strength. The strength of GABAergic inhibition can regulate the ability of excitatory synapses to undergo long-term plasticity, a key mechanism underlying the hippocampal circuit excitability and modifying the learning and memory process. Interestingly, a similar repetitive stimulation protocol used to induce LTP at glutamatergic can also trigger LTD at GABAergic synapses in CA1 pyramidal neurons [28, 29]. Such studies have allowed revealing the progress and consolidation of the epileptic stage as an abnormal form of long-term plasticity [15, 16], which seems to require simultaneous increase of excitatory and decrease of inhibitory neurotransmission.

## 2. Is the Glutamatergic Plasticity Different in Epileptic Brain Compared to Normal One?

Long-term changes in synaptic efficacy of glutamatergic neurotransmission are a most widely studied model of learning and memory [30]. Depending on stimulus trend, synaptic changes can increase or decrease the synaptic efficacy, leading to long-term potentiation (i.e., LTP) or depression (LTD), respectively. Experimentally, LTP results in a synaptic facilitation, lasting hours to months according to parameters and repetition of stimuli. In the hippocampal formation, one of brain structures involved in the storage of long-term memory and that seems to play a major role in declarative memory, the glutamatergic strengthening is activity-dependent and input-specific and requires temporal coincidence between pre- and postsynaptic depolarization due to voltage dependence of N-methyl-D-aspartate receptors (NMDARs) activation. The resulting calcium (Ca^2+^) influx can activate a plethora of signalling that promotes the protein synthesis, translocation of receptors, and gene expression, leading to structural and molecular long-lasting synaptic changes [30].

Several lines of evidences indicate that an abnormally enhanced glutamatergic activity, often referred to as the “*glutamate hypothesis*,” is one of the key alterations in the pathophysiology of epilepsy. Pioneering studies carried out in a chronic model of epilepsy both* in vivo* [18, 31] and* in vitro* [21, 22] showed that repeated electrical tetanizing stimulation produces burst of population spikes, whose duration and numbers progress with repetitive presentation of stimuli (see Box  1). Similarly, spontaneous recurrent seizures can be observed in other pharmacological epileptogenesis models (i.e., pilocarpine and pentylenetetrazol), which reproduces the repetitive neuronal activation evoked by stimulation [24, 25, 32]. In several models the progress of neural hyperexcitability is inhibited by NMDAR antagonists (i.e., APV and MK801). Interestingly, the progressive increasing of seizures is insensitive to APV once they were established, and their developing disrupts the hippocampal LTP [33, 34]. Like Hebbian synaptic plasticity, the activation of NMDARs is necessary to induce the long-term synaptic changes and developing synchronous firing, but not for the maintenance of epileptic seizures [22]. Overexpression of NMDARs and AMPARs in hippocampal formation has been widely documented both from resected tissue of TLE patients and in several animal epilepsy models [32, 35]. Indeed, the immunoreactivity patterns for AMPARs and NMDARs subunits GluR1, GluR2/3, or NR1 and NR2, respectively, showed alterations in all hippocampal subfields obtained from TLE patients, with differential distributions depending on subtype TLE (i.e., TLE sclerotic v/s TLE nonsclerotic) [36]. In particular, NR1 immunoreactivity was increased in the CA3-CA1* Stratum radiatum*, while GluR2/3 was expressed strongly in soma and proximal dendrites on both pyramidal neurons and dentate granule cells [32, 35]. It has been suggested that such expression as well as reorganization of the glutamate receptors is a feature of the epileptic hippocampus already remodelled. Like in NMDARs-dependent synaptic plasticity (i.e., LTP), these changes may provide one of the molecular substrates that supports the enhancement of glutamatergic activity in the pathophysiology of epileptogenesis. In addition, the most commonly used anticonvulsant drugs exert their effects by decreasing glutamatergic transmission and/or neuronal excitability (i.e., levetiracetam, oxcarbazepine, and lamotrigine) [37, 38] or by increasing GABAergic inhibition (i.e., vigabatrin, tiagabine, and valproate). Also, the induction of experimental epilepsy is inhibited by some drugs that bind selectively to proteins of neurotransmitter release machinery (i.e., levetiracetam), reducing the glutamatergic transmission, and is commonly used as antiepileptic [39].

Extracellular glutamate, measured by* in vivo* microdialysis, is elevated fivefold in the epileptogenic human hippocampus during interictal state and increases 30 times higher than normal during the seizure [40]. Moreover, the content as well as activity of glutamine synthase (GS), the enzyme responsible of glutamate-glutamine conversion within the astrocytes, is decreased in brain tissue of TLE patients [41, 42]. Indeed, L-methionine sulfoximine (MSO), a specific inhibitor of GS, is an effective seizure inductor [43], via reduction in the amplitude of the inhibitory GABA-mediated postsynaptic current (IPSC) in hippocampal neurons and changing the astroglial and/or the extracellular accumulation of glutamate. Two glial specific transporters, EAAT1 and EATT2, which are largely responsible for glutamate clearance from extracellular space, are expressed in astrocytes [44]. The inhibition of glial EATT2 induces epileptic bursts [45], while knockout mice for EAAT1/EAAT2 showed spontaneous epileptic seizures and profound hyperexcitability compared to wild type mice. [45]. In addition, in a drug-resistant form of human epilepsy, a reduced level of EAAT2 mRNA has been reported [46]. Conversely, no differences were detected in EAAT1 or EAAT2 expression levels between control and sclerotic (i.e., like TLE) human hippocampus [47, 48] suggesting the participation of additional nonmetabolic factors.

Other molecular targets that are overexpressed in both epileptic patients and experimental models are the metabotropic glutamate receptors (mGluRs). mGluRs form a family of eight subtypes, classified into three groups, where group I and group II include mGluR1/5 and mGluR2/3, respectively. These receptors are widely expressed in both neurons and astrocytes through the brain and have been implicated in the modulation of both glutamatergic and GABAergic neurotransmission as well as in glia-neuron crosstalk [49–51]. Indeed, we and others have recently demonstrated that the glutamate spontaneously released from astrocyte sets the basal probability of glutamate release via group I mGluRs activation [13, 52–54] and that their overactivation could be implicated in the glutamate upregulation on epileptic brain (see below).

These long-term changes in the expression/reorganization of the glutamate receptors, transporters, and/or metabolic enzymes represent plastic changes at synaptic level that contributes to progression and development of epilepsy. Like NMDARs-dependent synaptic plasticity (i.e., LTP), morphological and functional changes in the postsynaptic/presynaptic compartment and neuron-glia signalling would be providing one of the molecular substrates that supports the enhancement glutamatergic activity required to develop epilepsy.

## 3. Is the GABAergic Synaptic Plasticity Implicated in the Epilepsy?

The GABAergic interneurons play an essential role in the synchronization of local networks and functional coupling in different brain [55]. Given the crucial role of inhibitory synapses in regulating both neuronal excitability and excitatory synaptic plasticity, changes in GABAergic synaptic efficacy can have important functional and pathological consequences [56]. As in excitatory neurotransmission, changes in GABA (*γ*-aminobutyric acid) receptor composition, expression, cellular distribution, and function, therefore, have profound consequences for neural excitability, and they are associated with the etiology of several neurological and mental diseases, including epilepsy [57].

## 4. GABA Plasticity: Synaptic versus Extrasynaptic GABA Receptors, Synaptic Efficacy, and Epilepsy

The regulation of relative strengths of excitatory and inhibitory synapses is a powerful way to stabilize network activity. Synaptic communication requires constant adjustments of pre- and postsynaptic efficacies, to optimize their function and/or adapt to a changing environment [58, 59]. Research carried out during the last two decades has made it clear that inhibitory synapses undergo short- and long-term forms of synaptic plasticity [56, 60]. The activity-dependent changes in inhibitory neurotransmission are typically accompanied by alterations in GABAergic efficacy and synapse structure that range from morphological reorganization of postsynaptic density to* de novo* formation and elimination of inhibitory contacts [61]. Depending on the inhibitory interneuron cell type and the brain region, the inhibitory plasticity is dependent on changes in either GABA release or the number/sensitivity/responsiveness of postsynaptic GABA receptors [56]. Inhibitory neurotransmission in the mammalian brain is largely a result of GABA signaling. GABA acts on two main classes of receptors, the type-A ionotropic GABA receptors (GABA_A_Rs) and the type-B metabotropic GABA receptors (GABA_B_Rs). Similar to nicotinic receptors, GABA_A_Rs are composed of different subunits assembled in a pentameric structure [62–64]. Native heteropentameric GABA_A_Rs subtypes have a high structural diversity, being divided into classes based on sequence identity: *α*(1–6); *β*(1–3), *γ*(1–3), *δ*, *ε*, *π*, *θ*, and *ρ*(1–3). GABA_A_Rs comprising *γ*2 and *α* 1–3 subunits are most common type of receptor at synapses sites. These GABA_A_Rs are ligand-gated channels permeable to chloride and bicarbonate that produce minimal direct change in the membrane potential but generate a large conductance that shunts the excitatory depolarization [65]. Furthermore, the extrasynaptic GABA_A_Rs comprise *α*4 and *α*6 subunits combined to *δ* subunit, and they are responsible for tonic inhibition [66]. In addition to subunit composition and localization, other mechanisms exist that control GABA_A_Rs on a rapid time-scale, such as regulation of receptor trafficking, clustering, and surface expression. At synapses, GABA_A_Rs constitutively undergo significant rates of endocytosis, being rapidly recycled or targeted for lysosomal degradation [67, 68]. Therefore, changes in the rates of GABA_A_Rs endocytosis and/or endocytic sorting represent potentially powerful mechanisms to regulate GABA_A_Rs cell surface number and inhibitory synaptic transmission [67, 69]. A direct relationship between the number of postsynaptic GABA_A_Rs and the strength of the synapse has been demonstrated [70, 71]. Therefore, to maintain a stable cell-surface receptor number, continual membrane insertion of newly synthesized or recycled receptors is required [72]. Therefore, changes in the trafficking of these receptors could regulate neuronal plasticity and contribute to the manifestation of a wide range of brain disorders [72–74].* Postmortem* studies in epileptic patients have revealed severe alteration in the number and expression of extrasynaptic GABA_A_Rs [75]. The trafficking of GABA_A_Rs to and from the membrane is altered during prolonged seizures and has been suggested to contribute to benzodiazepine pharmacoresistance in patients with status epilepticus (SE) [72, 76]. Interestingly, the epileptiform activity alters intracellular Ca^2+^ concentrations and calcineurin activity, which correlates with the decrease of GABA_A_Rs from the surface, possibly contributing to pathological signaling during SE [77]. The loss of GABAergic interneurons and/or a reduction in the GABAergic synapses could result in a decrease of GABA release, a decrease of extracellular GABA availability, and a reduction of tonic inhibition. In rat hippocampal culture model it has observed a downregulation of tonic GABA inhibition after chronic epileptogenic stimulation [78]. On the other hand, experimental evidence indicates that, in epilepsy, at least epileptogenic period the tonic GABAergic current are maintained or augmented in several hippocampal neurons [79, 80]. Reduction of several subtypes of extra and perisynaptic GABA_A_Rs has been reported in hippocampus of animals with TLE. A molecular and pharmacological study shows that the overexpression of two subtypes of extrasynaptic GABA_A_Rs (*α*5*β*3*γ*2 and *α*6*β*3*δ*) can enhance the tonic inhibition and reduce the epileptiform activity [81]. In addition, mice lacking the GABA_A_Rs *δ* subunit exhibit an impaired GABAergic efficacy and increased seizure susceptibility, and mice lacking the GABA_A_Rs *α*5 subunit exhibit a diminished tonic inhibition and elevated hyperexcitability [82]. Therefore, the physiological consequences of these changes depend not only on the subunits of GABA_A_Rs, but also on somatodendritic localization, as well as the presynaptic, perisynaptic, or extrasynaptic sites composition of GABA_A_Rs [83].

The functional interaction between dendritic ionic channels and neurotransmitter receptors (i.e., GABA_A_Rs) plays a determinant role in neural integrating and dendritic excitability. Because of its characteristic biophysical properties, some cationic current, as noninactivating, mixed Na^+^-K^+^ current, Ih can shape both hyperpolarizing and depolarizing inputs. It has been demonstrated that after febrile seizures the dendritic Ih current is upregulated, which results in general enhancement of hippocampal dendritic excitability [84]. The febrile seizures induce a PKA-dependent presynaptic potentiation of GABAergic IPSC, GABA_A_Rs mediated IPSCs in CA1 pyramidal cells [5]. After febrile seizures the burst of IPSP can activate the Ih current inducing a postinhibitory rebound and can result in pyramidal-cell discharges following the inhibitory barrage, which can be prevented by application of selective h channel blocker ZD-7288 [5].

Recently, an endocannabinoids-dependent presynaptic long-term depression (LTD) has been described in different brain regions [28, 85, 86]. Typically, endocannabinoids (eCBs) can transiently or permanently reduce the GABA neurotransmission by activation of type 1 eCBs receptors (CB1Rs) [87–90]. Different pattern of neuronal activity can induce simultaneously LTP at excitatory glutamatergic synapses and presynaptic form of LTD at inhibitory GABAergic synapses in hippocampal CA1 PNs [29, 91]. Cannabinoids have been proposed as a “circuit breaker,” because of their ability to stop the progress of seizures and limit neuronal degeneration [92, 93]. After a brain insult that induces a cellular depolarization, glutamate release, and increase of intracellular Ca^2+^, neurons can release eCBs, which can damp the seizures and reduce the neuronal cell death as a consequence of SE. Several data obtained from both human and animal models of epilepsy showed changes in CB1R expression at hippocampal GABAergic synapses [94, 95]; alterations in the production and breakdown of eCBs could thus have profound effects on excitability and synaptic transmission in the hippocampus [93]. While eCBs are sufficiently powerful to silence a synapse, the activation of CB1Rs, generally confined to the synaptic axonal terminal [92, 96] (but see below), does not influence somatic action potential firing. As a result, excitation of dendrites can trigger repetitive somatic action potentials that readily travel to the synaptic terminals where they can reduce and/or eliminate the CB1R-mediated inhibition of release. By decreasing inhibition, the increase of CB1Rs on inhibitory terminals will increase the E/I ratio and shift the system toward hyperexcitability. Interestingly, upregulation of CB1Rs is itself dependent on CB1R activation during the seizures and can be prevented by CB1R antagonists [97]. In contrast, decreased activation of the CB1 receptor, through either genetic deletion of the receptor or treatment with a CB1 antagonist, can increase the pilocarpine seizure severity without modifying seizure-induced cell proliferation and cell death [98]. Recently, it has been shown that inhibition of hydrolase *α*/*β*-hydrolase domain 6 (ABHD6), which is involved in eCBs metabolism, can protect against seizures in mouse models of epilepsy [99].

In addition, CB1 receptor in specific neuronal has provided functional and anatomical evidence that CB1 receptors on hippocampal glutamatergic neurons are necessary for the CB1-dependent protection against acute excitotoxic seizures [100]. Dentate gyrus mossy glutamatergic cells, where CB1 receptors are present at low but detectable levels, are the central mediators of on-demand endocannabinoid-dependent protection against excitotoxicity seizures in the adult mouse brain [100]. Moreover the activation of CB1R present on glutamatergic terminals can suppress recurrent excitation in the dentate gyrus of mice with TLE, suggesting an anticonvulsive role of cannabinoids [101]. It has demonstrated that status epilepticus can selectively compromise GABA release at synapses from a subtype of hippocampal interneurons dentate accommodating interneurons to fast-spiking basket cells interneurons. The functional decrease in CB1R-sensitive inhibition of FS-BCs resulted from enhanced baseline GABA_B_Rs-mediated suppression of synaptic release after SE [102]. Recently, it has shown that block of monoacylglycerol lipase and the subsequent increase of 2-arachidonoylglycerol (2-AG) can delay the development of generalized seizures and decrease the seizures and postdischarge duration in the kindling model of TLE [103]. Taken together these data indicate that the endocannabinoids signaling might be a promising target to control neuronal excitability during seizure activity.

As we have highlighted before, GABA_A_Rs in the CNS mediate both fast synaptic and tonic inhibition. The phasic inhibition is characterized by a short-lasting inhibitory postsynaptic potential (IPSP) and tonic inhibition is characterized by persistent, long-lasting one (IPSP). GABA_A_Rs mediating tonic inhibition are different from those mediating phasic inhibition. They are located outside the synapse and hence are referred to as perisynaptic or extrasynaptic receptors [63]. The effect of extracellular GABA on high affinity, slowly desensitizing extrasynaptic GABA_A_Rs, is termed “tonic inhibition.” This tonic activation of extrasynaptic and perisynaptic GABA_A_Rs provides a powerful means of regulating neuronal excitability [79]. Several polymorphisms and mutations in genes encoding extrasynaptic GABA_A_Rs have been associated with several types of human epilepsies, implying that dysfunction of extrasynaptic GABA_A_R-mediated currents has dramatic effects on neuronal excitability [104, 105]. In addition, application of tiagabine (or EF1502), a GABA transporter inhibitor, enhanced the anticonvulsant effect of GABA_A_Rs agonist gaboxadol [106]. The tonic inhibition mediated by extrasynaptic GABA_A_Rs is dependent on the GABA availability, whose modification may play a prominent role during SE. Tonic GABAergic signaling, extracellular GABA availability, and inactivation of GABA neurotransmission are highly sensitive to changes in the efficacy of GABA uptake transporter (GATs 1–4) located in the presynaptic nerve ending as well as in astrocytic processes ensheathing synapses [106, 107]. Also, recaptured GABA by the axon terminals is mostly reused to fill vesicles via vesicular GAT [108]. According to their essential function within the control of synaptic and extrasynaptic GABA levels, GATs have been linked to epilepsy [109]. Drugs acting either selectively or nonselectively at GATs are used for antiepileptic medication [110, 111]. GAT-1 inhibitors are effective against the kindled focal and secondary generalized seizures [110].

Astrocytes can set the tone of GABAergic inhibition in local neural circuits [63]. In the neocortex, GAT-1 and GAT-3 are the most abundantly expressed ones, with GAT-1 mainly expressed in GABAergic interneurons and less on astrocytes, while GAT-3 is mainly expressed in astrocytes [112]. Recent works show that astrocytic GAT-3 is important to control the excitability of hippocampal cells when network activity is increased [112]. Several studies showed that astrocytes can release GABA and activate extrasynaptic high affinity GABA receptor to mediate tonic inhibition in neighboring neurons and modulate the brain physiology [113].

As we already highlighted, in adult brain the activation of GABA_A_R causes neuronal membrane hyperpolarization due to increased chloride permeability. This hyperpolarizing response critically depends on chloride extrusion via the K-Cl-cotransporter KCC2. The role of KCC2 is critical in order to maintain the equilibrium potential of GABA (*E*_GABA_) at a sufficiently negative level to prevent the neuron from firing action potentials [114, 115]. The downregulation of KCC2 in response to trauma and/or intense seizure activity leads to a long-lasting decrease in the efficacy of both shunting and voltage inhibition and results in the development of network hyperexcitability. Decreased KCC2-mediated chloride extrusion and impaired hyperpolarizing GABA_A_R-mediated currents have been implicated in TLE, as well as other types of epilepsy [116, 117]. Seizure-induced downregulation of KCC2 activity depends on posttranscriptional mechanisms [115, 118] including protein phosphatase 1-mediated dephosphorylation of KCC2 at serine 9 and cleavage by the protease calpain, which is activated by Ca^2+^ and/or BDNF [115].

On the other hand, functional GABA_B_Rs are formed by heterodimeric assembly of two subunits GABA_B_1 and GABA_B_2 [119, 120]. GABA_B_Rs are coupled to different effectors via GTP binding protein [121]. Postsynaptic GABA_B_Rs are coupled to Gi subtype of G-protein protein which downregulated cyclic AMP production and promoted activation of inwardly rectifying potassium channels resulting in a slow and sustained neuronal hyperpolarization [122]. Presynaptically located GABA_B_Rs inhibit transmitter release by inhibiting activation of voltage-gated Ca^2+^ channels [123, 124]. Several studies have shown that GABA_B_Rs can modulate cell survival, migration, and neuronal differentiation, as well as regulating synaptogenesis, maturation, and plasticity of synaptic connections [120]. GABA_B_Rs are essential for the stability of cortical network activity [125]. Thus, high doses of GABA_B_Rs antagonist disrupt the normal hippocampal and cortical oscillations including delta waves and sleep spindles, as well as fast gamma oscillations, and lead to epileptiform activity [126]. Also, GABA_B_Rs knock-out mice are prone to developing spontaneous seizures [127, 128]. Moreover, the GABA_B_R agonist baclofen can also promote excitability and seizure generation in both human patients and epilepsy animal models [129]. In addition, it has been shown that GABA_B_R expression is altered in both TLE patients and animal models [130, 131].

The persistent GABA_B_R activation in epileptic mice can suppress the inhibitory output from hippocampal cholecystokinin basket cell interneurons, which leads to disinhibition in hippocampal networks, enhances gamma activity, and promotes the transition to pathological hyperexcitability These data suggest an important role of GABA_B_Rs in the generation and control of epileptiform activity and act as a promising therapeutic target for the treatment of seizures.

In parallel to functional changes, multiple morphological changes are found in human and animal epilepsy models. Axonal sprouting of excitatory and inhibitory neurons of hippocampal formation is frequently observed in temporal lobe epilepsy [132, 133]. The loss of GABAergic interneurons and compensatory axonal sprouting are the main inhibitory reasons for GABAergic neuron decrease, restoration, and potentiation. The inhibitory neurons exhibit similar axonal growth and synaptogenesis, which has been suggested as an explanation for the persistence or increase in labeling of GABAergic axons and terminals in human temporal lobe epilepsy and related animal models [134, 135]. It has been observed that hippocampal SOM/GABA neurons can undergo substantial axonal reorganization, project beyond their normal innervation territory, and form functional but aberrant circuitry in a mouse model of epilepsy [135, 136]. Recently, in a rat model of TLE it has showed a loss of CCK-containing GABAergic terminals and synapses in the inner molecular layer of the dentate gyrus causes the reduction of CCK-containing GABAergic synaptic transmission to DGCs, tending to reduce seizure threshold [137].

## 5. Astrocyte: The Third Element in the Abnormal Plasticity of Epilepsy

It is well known that astrocytes form a “tripartite” functional unit with presynaptic and postsynaptic structures, which regulates synaptic transmission and neuronal plasticity [13, 138]. This astrocyte-neuron communication allows that Ca^2+^-dependent glutamate release from astrocytes can increase the glutamatergic neurotransmission through metabotropic glutamate receptors (mGluRs) activation located in the presynaptic terminal [13, 139–141]. While brain disease mechanisms are largely considered to have a neuronal origin, growing evidence suggests that disturbances of astrocyte-neuron cross-talk are related to brain disorders including epilepsy [54, 142–144]. As consequence of neuropathological conditions including epilepsy, reactive astrocytes exhibit several changes in the expression rate of proteins, including cytoskeleton proteins, transporters, enzymes, and receptors. Moreover, proinflammatory molecules can induce the releases ATP from microglia, which, via gliotransmitter release from neighbours astrocyte, modify the synaptic efficacy [145].

Several evidences suggest that mGluRs would be a molecular key in the alteration of synaptic plasticity in an epileptic network, where glutamate-mediated gliotransmission is a putative signal that contributes to the increased excitability and neuronal hypersynchronicity [146].

Overexpression of mGluR group I/II in reactive astrocytes and neurons in hippocampal tissue from both TLE patients and epilepsy experimental models has been widely reported [51, 146, 147]. These findings have been also corroborated in a kainate-induced model of epilepsy, in which mGluRs are also overexpressed and colocalized in hippocampal GFAP-positive astrocytes [148]. The kindling-induced enhancement of LTP and maintenance population spike was prevented in presence of specific mGluR group I antagonists [149, 150]. In acute epilepsy model increasing of the astrocytic Ca^2+^ waves correlates with increase in frequency of synchronic neuronal depolarizations [151]. This TTX-insensitive increase in astrocytic Ca^2+^ wave preceded or occurred concomitantly with paroxysmal depolarization shift (PDS). Moreover, several anticonvulsive agents potently reduced astrocytic Ca^2+^ signalling and removed the epileptic activity [152]. Interestingly, this epileptic activity was inhibited by the application of antagonists NMDARs and AMPARs providing concrete evidence about the role played by the astrocytes as a new source of glutamatergic excitation to epileptic activity. Taken together, glutamate release from astrocyte has been implicated in the glutamatergic imbalance described in epileptic networks, maintaining a high glutamatergic tone and setting excitatory transmission near to seizure threshold [40, 144]. Recently, we showed that astrocytes from epileptic hippocampus display Ca^2+^-dependent hyperexcitability, through a mechanism that requires the activation of astroglial P2Y1R which increases glutamate-mediated gliotransmission, upregulating the synaptic efficacy in the CA3-CA1 circuit via presynaptic mGluR5 activation [54]. At postsynaptic level, glutamate release from astrocytes induces slow inward current (SIC) in adjacent neurons, mediated by extrasynaptic NMDARs activation [138, 153]. The functional role of SICs is involved in the synchronicity of neuronal networks due to their capacity to induce SIC-dependent depolarization in pyramidal neurons distant by ~100 *μ*m, which would allow for simultaneously controlling the excitability at a group of neighboring pyramidal cells [151, 154]. Several evidences have described that an increase of astrocytic Ca^2+^ transients during acute epileptiform activities is correlated to an increase in frequency of SIC [153, 155] and preceded or occurred concomitantly with paroxysmal depolarization shift (PDS) [152]. However, the SIC contribution in the hypersynchronic neuronal discharges that characterize the ictal-interictal activities is still unknown.

Recently, it has been demonstrated that astrocytes are involved in the eCBs system, responding to exogenous cannabinoids ligands as well as eCBs through activation of CB1R [156]. This activation increased the astrocytes Ca^2+^ levels through the mobilization of Ca^2+^ from internal stores and stimulates the release of glutamate that modulates synaptic transmission and plasticity. While a study reported a proconvulsive effect of cannabinoids ligands, another showed that activation of CB1Rs have a potent antiepileptic activity [97, 157]. However, a recent report has shown that CB1R antagonist reduces the maintenance of epileptic discharges, which can be abolished when the intracellular astrocyte Ca^2+^ increase is prevented [158] suggesting that gliotransmission triggered by astroglial Ca^2+^ elevation is involved in the hippocampal epileptic activity.

The role of astrocyte in modulation of GABAergic transmission is less understood. Like glutamate release from neurones, GABA also evokes Ca^2+^ oscillations in astrocytes via GABA_B_ receptors [159]. Glutamate release from astrocytes can mediate either depression or potentiation [160, 161] of inhibitory transmission, contributing to E/I imbalance on the projection neurons. In particular, varied mechanisms can contribute to glutamate depressor effects on GABAergic interneurons, including decrease of amplitude of miniature IPSC and action potential-dependent GABA release by kainate receptors activation [161]. Also it has been demonstrated that activation of presynaptic mGluR group III can depress the GABAergic transmission to identify interneurons [162–164] as well as to pyramidal cells of hippocampus [165].

**Figure 1:**
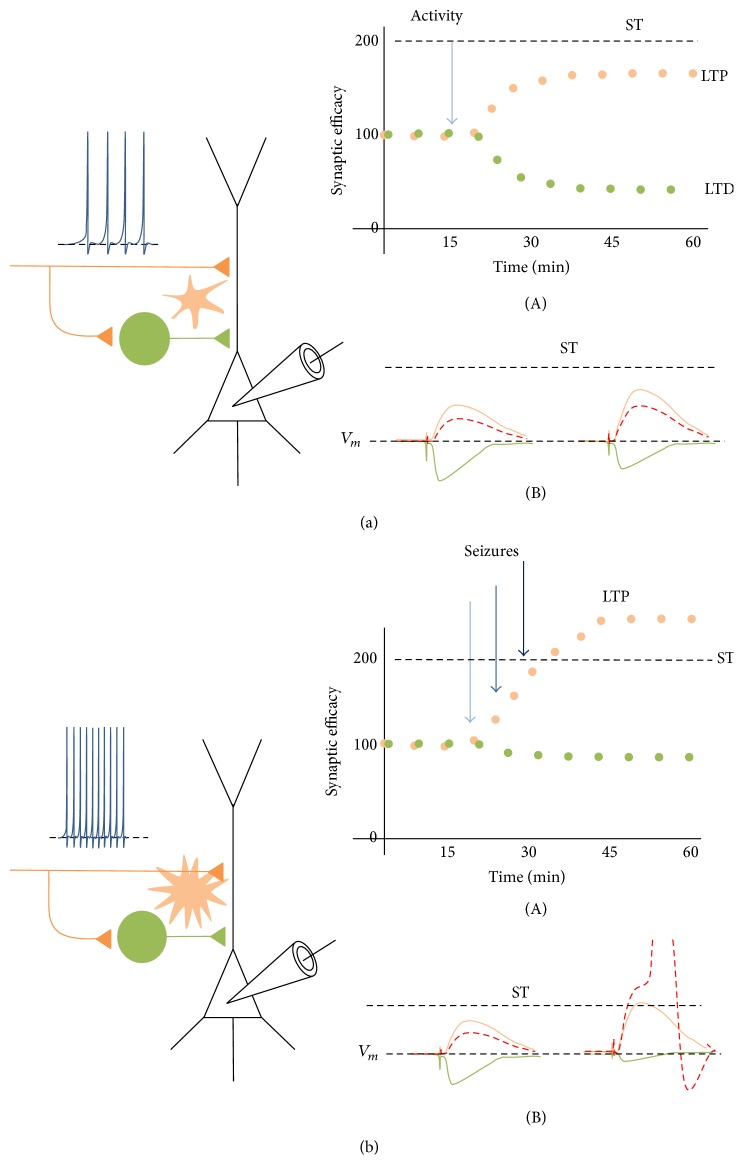
Glutamatergic and GABAergic long-term plasticity and tripartite neuronal-astroglial network in normal and epileptic brain. (a) During physiological neuronal activity, coincidence between postsynaptic depolarization and glutamatergic (orange) and GABAergic interneuron (green) simultaneously activated induces increase of synaptic efficacy (i.e., mean amplitude of postsynaptic response) in both glutamatergic [CB1] synapses (LTP) at the same time of a decrease of efficacy of GABAergic transmission (LTD; A). (B) By GABAergic and glutamatergic input integration, the net increase in membrane potential falls below the seizure threshold (ST). (b) During epileptiform neuronal activity, astroglial hyperexcitation through GABA_A_Rs, GluRs, and/or eCBsRs activation, which increases the intracellular Ca^2+^ release of astroglial glutamate, increasing the excitatory neurotransmission while inhibitory transmission remains unchanged (A). (B) In this condition, glutamatergic/GABAergic rate results in an excitatory imbalance, exceeding the seizure threshold.

These evidences suggest that the activation of presynaptic mGluR group III in GABAergic presynaptic terminals added to activation of presynaptic mGluR group I in glutamatergic presynaptic terminals may be, at least in part, explaining the simultaneous E/I imbalance exhibited in epileptic brain (Figure 1). Other gliotransmitters released from astrocytes also have been associated with changes in the synaptic efficacy and excitability in E/I circuit. ATP increased astroglial Ca^2+^ elevations and depolarized the GABAergic interneurons, enhancing the inhibition onto projections neurons of hippocampus [12, 166]. Similarly, D-serine released from astrocytes controls the NMDA receptor-mediated synaptic potentiation. Because astrocyte-neuron communication is a form of communication cell and synapse specific, astrocyte may represent glutamatergic sources to modulate the E/I balance. However, it is unknown if astroglial glutamate, ATP, or D-serine can simultaneously regulate the glutamatergic and GABAergic plasticity.

## 6. Conclusions and Future Directions

The cellular basis of learning and memory is believed to depend on short- and long-lasting changes in synaptic plasticity. Typically, changes in the strength and plasticity of excitatory synaptic transmission have been assumed to underlie learning and memory processes. More recent investigation has demonstrated that inhibitory transmission is not only plastic; it also modulates the efficacy and threshold of excitatory synaptic plasticity. In several brain areas, the excitatory and inhibitory synaptic plasticity can occur simultaneously [29, 56]. The same patterns of activation that trigger LTP in excitatory synapses can also induce short- or long-lasting plasticity in inhibitory synapses. This functional balance between excitatory and inhibitory synapses is established during development and maintained throughout life and is essential to brain function [114]. The astrocytes are strategically positioned, close to excitatory and inhibitory synapses, allowing them to integrate the adjacent synaptic activity via gliotransmitters release, including control of synchronous depolarization of groups of neurons [153], reducing the threshold of synaptic plasticity or suppressing the synaptic transmission [13, 81]. Through these coordinating actions the astrocytes can contribute to the excitatory/inhibitory balance, modulating the neural network operations in a specific-cells manner.

As in a variety of neural disorders, deregulation of the E/I balance of synaptic transmission has been associated with epilepsy. It is still unknown whether this imbalance is causative for the disease or a consequence of pathological pattern of network activity. Even though the long-lasting molecular changes that lead to LTP/LTD in normal brain seem to be the same required to turn into an epileptic one, astrocyte-neurons networks represent a new pathological key to explain the concerted alterations in synaptic plasticity to generate the E/I imbalance. Therefore, future experimental approaches should give consideration to such astroglial-neuronal network in the brain, which may offer new therapeutic target for treatment of nervous system disorders.

## References

[1] Gastaut H. (1973). Letter: Epileptic seizures. *Developmental Medicine and Child Neurology*.

[2] Moghim N., Corne D. W. (2014). Predicting epileptic seizures in advance. *PLoS ONE*.

[3] Meldrum B. S. (1992). Excitatory amino acids in epilepsy and potential novel therapies. *Epilepsy Research*.

[4] Guerriero R. M., Giza C. C., Rotenberg A. (2015). Glutamate and GABA imbalance following traumatic brain injury. *Current Neurology and Neuroscience Reports*.

[5] Chen K., Aradi I., Thon N., Eghbal-Ahmadi M., Baram T. Z., Soltesz I. (2001). Persistently modified h-channels after complex febrile seizures convert the seizure-induced enhancement of inhibition to hyperexcitability. *Nature Medicine*.

[6] Khazipov R., Valeeva G., Khalilov I. (2015). Depolarizing GABA and developmental epilepsies. *CNS Neuroscience and Therapeutics*.

[7] Kwan P., Li H. M., Al-Jufairi E. (2010). Association between temporal lobe P-glycoprotein expression and seizure recurrence after surgery for pharmacoresistant temporal lobe epilepsy. *Neurobiology of Disease*.

[8] McNamara J. O., Huang Y. Z., Leonard A. S. (2006). Molecular signaling mechanisms underlying epileptogenesis. *Science’s STKE*.

[9] Morimoto K., Fahnestock M., Racine R. J. (2004). Kindling and status epilepticus models of epilepsy: rewiring the brain. *Progress in Neurobiology*.

[10] Weaver D. F. (2003). Epileptogenesis, ictogenesis and the design of future antiepileptic drugs. *Canadian Journal of Neurological Sciences*.

[11] Scharfman H. E. (2007). The neurobiology of epilepsy. *Current Neurology and Neuroscience Reports*.

[12] Bowser D. N., Khakh B. S. (2004). ATP excites interneurons and astrocytes to increase synaptic inhibition in neuronal networks. *The Journal of Neuroscience*.

[13] Bonansco C., Couve A., Perea G., Ferradas C. Á., Roncagliolo M., Fuenzalida M. (2011). Glutamate released spontaneously from astrocytes sets the threshold for synaptic plasticity. *European Journal of Neuroscience*.

[14] Henneberger C., Papouin T., Oliet S. H. R., Rusakov D. A. (2010). Long-term potentiation depends on release of D-serine from astrocytes. *Nature*.

[15] Cain D. P. (1989). Long-term potentiation and kindling: how similar are the mechanisms?. *Trends in Neurosciences*.

[16] Meador K. J. (2007). The basic science of memory as it applies to epilepsy. *Epilepsia*.

[17] Morales J. C., Álvarez-Ferradas C., Roncagliolo M. (2014). A new rapid kindling variant for induction of cortical epileptogenesis in freely moving rats. *Frontiers in Cellular Neuroscience*.

[18] Racine R. J. (1972). Modification of seizure activity by electrical stimulation. II. Motor seizure. *Electroencephalography and Clinical Neurophysiology*.

[19] Goddard G. V., McIntyre D. C., Leech C. K. (1969). A permanent change in brain function resulting from daily electrical stimulation. *Experimental Neurology*.

[20] Dhir A. (2012). Pentylenetetrazol (PTZ) kindling model of epilepsy. *Current Protocols in Neuroscience*.

[21] Stasheff S. F., Bragdon A. C., Wilson W. A. (1985). Induction of epileptiform activity in hippocampal slices by trains of electrical stimuli. *Brain Research*.

[22] Stasheff S. F., Anderson W. W., Clark S., Wilson W. A. (1989). NMDA antagonists differentiate epileptogenesis from seizure expression in an in vitro model. *Science*.

[23] Slater N. T., Stelzer A., Galvan M. (1985). Kindling-like stimulus patterns induce epileptiform discharges in the guinea pig in vitro hippocampus. *Neuroscience Letters*.

[24] Mazzuferi M., Kumar G., Rospo C., Kaminski R. M. (2012). Rapid epileptogenesis in the mouse pilocarpine model: video-EEG, pharmacokinetic and histopathological characterization. *Experimental Neurology*.

[25] Dhir A. (2012). UNIT 9.37 Pentylenetetrazol (PTZ) kindling model of epilepsy. *Current Protocols in Neuroscience*.

[26] Taylor C. P., Dudek F. E. (1982). Synchronous neural afterdischarges in rat hippocampal slices without active chemical synapses. *Science*.

[27] Malenka R. C., Bear M. F. (2004). LTP and LTD: an embarrassment of riches. *Neuron*.

[28] Chevaleyre V., Castillo P. E. (2003). Heterosynaptic LTD of hippocampal GABAergic synapses: a novel role of endocannabinoids in regulating excitability. *Neuron*.

[29] Ahumada J., Fernández de Sevilla D., Couve A., Buño W., Fuenzalida M. (2013). Long-term depression of inhibitory synaptic transmission induced by spike-timing dependent plasticity requires coactivation of endocannabinoid and muscarinic receptors. *Hippocampus*.

[30] Malenka R. C. (2003). The long-term potential of LTP. *Nature Reviews Neuroscience*.

[31] Goddard G. V. (1967). Development of epileptic seizures through brain stimulation at low intensity. *Nature*.

[32] Lopes M. W., Soares F. M. S., De Mello N. (2013). Time-dependent modulation of AMPA receptor phosphorylation and mRNA expression of NMDA receptors and glial glutamate transporters in the rat hippocampus and cerebral cortex in a pilocarpine model of epilepsy. *Experimental Brain Research*.

[33] Schubert M., Siegmund H., Pape H.-C., Albrecht D. (2005). Kindling-induced changes in plasticity of the rat amygdala and hippocampus. *Learning and Memory*.

[34] Zhu G., Liu Y., Wang Y., Bi X., Baudry M. (2015). Different patterns of electrical activity lead to long-term potentiation by activating different intracellular pathways. *Journal of Neuroscience*.

[35] Naylor D. E., Liu H., Niquet J., Wasterlain C. G. (2013). Rapid surface accumulation of NMDA receptors increases glutamatergic excitation during status epilepticus. *Neurobiology of Disease*.

[36] Mathern G. W., Pretorius J. K., Leite J. P. (1998). Hippocampal AMPA and NMDA mRNA levels and subunit immunoreactivity in human temporal lobe epilepsy patients and a rodent model of chronic mesial limbic epilepsy. *Epilepsy Research*.

[37] Stief F., Zuschratter W., Hartmann K., Schmitz D., Draguhn A. (2007). Enhanced synaptic excitation-inhibition ratio in hippocampal interneurons of rats with temporal lobe epilepsy. *European Journal of Neuroscience*.

[38] Landmark C. J. (2007). Targets for antiepileptic drugs in the synapse. *Medical Science Monitor*.

[39] Sugaya Y., Maru E., Kudo K., Shibasaki T., Kato N. (2010). Levetiracetam suppresses development of spontaneous EEG seizures and aberrant neurogenesis following kainate-induced status epilepticus. *Brain Research*.

[40] Cavus I., Kasoff W. S., Cassaday M. P. (2005). Extracellular metabolites in the cortex and hippocampus of epileptic patients. *Annals of Neurology*.

[41] Eid T., Thomas M. J., Spencer D. D. (2004). Loss of glutamine synthetase in the human epileptogenic hippocampus: possible mechanism for raised extracellular glutamate in mesial temporal lobe epilepsy. *The Lancet*.

[42] Steffens M., Huppertz H.-J., Zentner J., Chauzit E., Feuerstein T. J. (2005). Unchanged glutamine synthetase activity and increased NMDA receptor density in epileptic human neocortex: implications for the pathophysiology of epilepsy. *Neurochemistry International*.

[43] Rowe W. B., Meister A. (1970). Identification of L-methionine-S-sulfoximine as the convulsant isomer of methionine sulfoximine. *Proceedings of the National Academy of Sciences of the United States of America*.

[44] Lehre K. P., Danbolt N. C. (1998). The number of glutamate transporter subtype molecules at glutamatergic synapses: chemical and stereological quantification in young adult rat brain. *The Journal of Neuroscience*.

[45] Tanaka K., Watase K., Manabe T. (1997). Epilepsy and exacerbation of brain injury in mice lacking the glutamate transporter GLT-1. *Science*.

[46] Proper E. A., Hoogland G., Kappen S. M. (2002). Distribution of glutamate transporters in the hippocampus of patients with pharmaco-resistant temporal lobe epilepsy. *Brain*.

[47] Cohen I., Navarro V., Le Duigou C., Miles R. (2003). Mesial temporal lobe epilepsy: a pathological replay of developmental mechanisms?. *Biology of the Cell*.

[48] Bjørnsen L. P., Eid T., Holmseth S., Danbolt N. C., Spencer D. D., de Lanerolle N. C. (2007). Changes in glial glutamate transporters in human epileptogenic hippocampus: inadequate explanation for high extracellular glutamate during seizures. *Neurobiology of Disease*.

[49] Eddleston M., Mucke L. (1993). Molecular profile of reactive astrocytes—implications for their role in neurologic disease. *Neuroscience*.

[50] Pin J. P., Duvoisin R. (1995). The metabotropic glutamate receptors: structure and functions. *Neuropharmacology*.

[51] Aronica E., van Vliet E. A., Mayboroda O. A., Troost D., Lopes Da Silva F. H., Gorter J. A. (2000). Upregulation of metabotropic glutamate receptor subtype mGluR3 and mGluR5 in reactive astrocytes in a rat model of mesial temporal lobe epilepsy. *European Journal of Neuroscience*.

[52] Panatier A., Vallée J., Haber M., Murai K. K., Lacaille J.-C., Robitaille R. (2011). Astrocytes are endogenous regulators of basal transmission at central synapses. *Cell*.

[53] De Pittà M., Volman V., Berry H., Ben-Jacob E. (2011). A tale of two stories: astrocyte regulation of synaptic depression and facilitation. *PLoS Computational Biology*.

[54] Álvarez-Ferradas C., Morales J. C., Wellmann M. (2015). Enhanced astroglial Ca^2+^ signaling increases excitatory synaptic strength in the epileptic brain. *Glia*.

[55] Mann E. O., Paulsen O. (2007). Role of GABAergic inhibition in hippocampal network oscillations. *Trends in Neurosciences*.

[56] Castillo P. E., Chiu C. Q., Carroll R. C. (2011). Long-term plasticity at inhibitory synapses. *Current Opinion in Neurobiology*.

[57] Schipper S., Aalbers M. W., Rijkers K. (2015). Tonic GABA_A_ receptors as potential target for the treatment of temporal lobe epilepsy. *Molecular Neurobiology*.

[58] Feldman D. E. (2012). The spike-timing dependence of plasticity. *Neuron*.

[59] Nelson S. B., Turrigiano G. G. (2008). Strength through diversity. *Neuron*.

[60] Gaiarsa J.-L., Caillard O., Ben-Ari Y. (2002). Long-term plasticity at GABAergic and glycinergic synapses: mechanisms and functional significance. *Trends in Neurosciences*.

[61] Vithlani M., Terunuma M., Moss S. J. (2011). The dynamic modulation of GABA(A) receptor trafficking and its role in regulating the plasticity of inhibitory synapses. *Physiological Reviews*.

[62] Costa E. (1998). From GABA_A_ receptor diversity emerges a unified vision of GABAergic inhibition. *Annual Review of Pharmacology and Toxicology*.

[63] Farrant M., Nusser Z. (2005). Variations on an inhibitory theme: phasic and tonic activation of GABA_A_ receptors. *Nature Reviews Neuroscience*.

[64] Sieghart W. (2006). Structure, pharmacology, and function of GABAA receptor subtypes. *Advances in Pharmacology*.

[65] Pouille F., Scanziani M. (2001). Enforcement of temporal fidelity in pyramidal cells by somatic feed-forward inhibition. *Science*.

[66] Brickley S. G., Mody I. (2012). Extrasynaptic GABA_A_ receptors: their function in the CNS and implications for disease. *Neuron*.

[67] Kittler J. T., Arancibia-Carcamo I. L., Moss S. J. (2004). Association of GRIP1 with a GABA_A_ receptor associated protein suggests a role for GRIP1 at inhibitory synapses. *Biochemical Pharmacology*.

[68] Jacob T. C., Moss S. J., Jurd R. (2008). GABA_A_ receptor trafficking and its role in the dynamic modulation of neuronal inhibition. *Nature Reviews Neuroscience*.

[69] Cuitino L., Godoy J. A., Farías G. G. (2010). Wnt-5a modulates recycling of functional GABAA receptors on hippocampal neurons. *Journal of Neuroscience*.

[70] Nusser Z., Hájost N., Somogyi P., Mody I. (1998). Increased number of synaptic GABA(A) receptors underlies potentiation at hippocampal inhibitory synapses. *Nature*.

[71] Nusser Z., Cull-Candy S., Farrant M. (1997). Differences in synaptic GABA_A_ receptor number underlie variation in GABA mini amplitude. *Neuron*.

[72] Naylor D. E., Liu H., Wasterlain C. G. (2005). Trafficking of GABA(A) receptors, loss of inhibition, and a mechanism for pharmacoresistance in status epilepticus. *The Journal of Neuroscience*.

[73] Brambilla P., Perez J., Barale F., Schettini G., Soares J. C. (2003). GABAergic dysfunction in mood disorders. *Molecular Psychiatry*.

[74] Tunnicliff G., Malatynska E. (2003). Central GABAergic systems and depressive illness. *Neurochemical Research*.

[75] Loup F., Picard F., Yonekawa Y., Wieser H.-G., Fritschy J.-M. (2009). Selective changes in GABA_A_ receptor subtypes in white matter neurons of patients with focal epilepsy. *Brain*.

[76] Goodkin H. P., Joshi S., Mtchedlishvili Z., Brar J., Kapur J. (2008). Subunit-specific trafficking of GABA_A_ receptors during status epilepticus. *The Journal of Neuroscience*.

[77] Eckel R., Szulc B., Walker M. C., Kittler J. T. (2015). Activation of calcineurin underlies altered trafficking of *α*2 subunit containing GABA_A_ receptors during prolonged epileptiform activity. *Neuropharmacology*.

[78] Qi J.-S., Yao J., Fang C., Luscher B., Chen G. (2006). Downregulation of tonic GABA currents following epileptogenic stimulation of rat hippocampal cultures. *Journal of Physiology*.

[79] Pavlov I., Walker M. C. (2013). Tonic GABA_A_ receptor-mediated signalling in temporal lobe epilepsy. *Neuropharmacology*.

[80] Caraiscos V. B., Elliott E. M., You-Ten K. E. (2004). Tonic inhibition in mouse hippocampal CA1 pyramidal neurons is mediated by *α*5 subunit-containing *γ*-aminobutyric acid type A receptors. *Proceedings of the National Academy of Sciences of the United States of America*.

[81] Sun Y., Wu Z., Kong S. (2013). Regulation of epileptiform activity by two distinct subtypes of extrasynaptic GABAA receptors. *Molecular Brain*.

[82] Glykys J., Mody I. (2006). Hippocampal network hyperactivity after selective reduction of tonic inhibition in GABA_A_ receptor *α*5 subunit-deficient mice. *Journal of Neurophysiology*.

[83] Glykys J., Mody I. (2007). Activation of GABA_A_ receptors: views from outside the synaptic cleft. *Neuron*.

[84] Chen K., Aradi I., Santhakumar V., Soltesz I. (2002). H-channels in epilepsy: new targets for seizure control?. *Trends in Pharmacological Sciences*.

[85] Chevaleyre V., Takahashi K. A., Castillo P. E. (2006). Endocannabinoid-mediated synaptic plasticity in the CNS. *Annual Review of Neuroscience*.

[86] Chávez A. E., Chiu C. Q., Castillo P. E. (2010). TRPV1 activation by endogenous anandamide triggers postsynaptic long-term depression in dentate gyrus. *Nature Neuroscience*.

[87] Wilson R. I., Kunos G., Nicoll R. A. (2001). Presynaptic specificity of endocannabinoid signaling in the hippocampus. *Neuron*.

[88] Ohno-Shosaku T., Shosaku J., Tsubokawa H., Kano M. (2002). Cooperative endocannabinoid production by neuronal depolarization and group I metabotropic glutamate receptor activation. *European Journal of Neuroscience*.

[89] Edwards D. A., Kim J., Alger B. E. (2006). Multiple mechanisms of endocannabinoid response initiation in hippocampus. *Journal of Neurophysiology*.

[90] Chevaleyre V., Heifets B. D., Kaeser P. S., Südhof T. C., Purpura D. P., Castillo P. E. (2007). Endocannabinoid-mediated long-term plasticity requires cAMP/PKA signaling and RIM1*α*. *Neuron*.

[91] Chevaleyre V., Castillo P. E. (2004). Endocannabinoid-mediated metaplasticity in the hippocampus. *Neuron*.

[92] Katona I., Freund T. F. (2008). Endocannabinoid signaling as a synaptic circuit breaker in neurological disease. *Nature Medicine*.

[93] Soltesz I., Alger B. E., Kano M. (2015). Weeding out bad waves: towards selective cannabinoid circuit control in epilepsy. *Nature Reviews Neuroscience*.

[94] Maglóczky Z., Tóth K., Karlócai R. (2010). Dynamic changes of CB1-receptor expression in hippocampi of epileptic mice and humans. *Epilepsia*.

[95] Karlócai M. R., Tóth K., Watanabe M. (2011). Redistribution of CB1 cannabinoid receptors in the acute and chronic phases of pilocarpine-induced epilepsy. *PLoS ONE*.

[96] Kawamura Y., Fukaya M., Maejima T. (2006). The CB1 cannabinoid receptor is the major cannabinoid receptor at excitatory presynaptic sites in the hippocampus and cerebellum. *The Journal of Neuroscience*.

[97] Wallace M. J., Wiley J. L., Martin B. R., DeLorenzo R. J. (2001). Assessment of the role of CB_1_ receptors in cannabinoid anticonvulsant effects. *European Journal of Pharmacology*.

[98] Kow R. L., Jiang K., Naydenov A. V., Le J. H., Stella N., Nathanson N. M. (2014). Modulation of pilocarpine-induced seizures by cannabinoid receptor 1. *PLoS ONE*.

[99] Naydenov A. V., Horne E. A., Cheah C. S. (2014). ABHD6 blockade exerts antiepileptic activity in PTZ-induced seizures and in spontaneous seizures in R6/2 mice. *Neuron*.

[100] Marsicano G., Goodenough S., Monory K. (2003). CB1 cannabinoid receptors and on-demand defense against excitotoxicity. *Science*.

[101] Bhaskaran M. D., Smith B. N. (2010). Cannabinoid-mediated inhibition of recurrent excitatory circuitry in the dentate gyrus in a mouse model of temporal lobe epilepsy. *PLoS ONE*.

[102] von Rüden E., Bogdanovic R., Wotjak C., Potschka H. (2015). Inhibition of monoacylglycerol lipase mediates a cannabinoid 1-receptor dependent delay of kindling progression in mice. *Neurobiology of Disease*.

[103] Yu J., Proddutur A., Swietek B., Elgammal F. S., Santhakumar V. (2015). Functional reduction in cannabinoid-sensitive heterotypic inhibition of dentate basket cells in epilepsy: impact on network rhythms. *Cerebral Cortex*.

[104] Dibbens L. M., Feng H.-J., Richards M. C. (2004). GABRD encoding a protein for extra- or peri-synaptic GABAA receptors is a susceptibility locus for generalized epilepsies. *Human Molecular Genetics*.

[105] Eugène E., Depienne C., Baulac S. (2007). GABAA receptor *γ*2 subunit mutations linked to human epileptic syndromes differentially affect phasic and tonic inhibition. *Journal of Neuroscience*.

[106] Schousboe A., Madsen K. K., Barker-Haliski M. L., White H. S. (2014). The GABA synapse as a target for antiepileptic drugs: a historical overview focused on GABA transporters. *Neurochemical Research*.

[107] Durkin M. M., Smith K. E., Borden L. A., Weinshank R. L., Branchek T. A., Gustafson E. L. (1995). Localization of messenger RNAs encoding three GABA transporters in rat brain: an in situ hybridization study. *Molecular Brain Research*.

[108] Zhou Y., Danbolt N. C. (2013). GABA and glutamate transporters in brain. *Frontiers in Endocrinology*.

[109] Sharopov S., Chen R., Sun H. (2014). Inhibition of different GABA transporter systems is required to attenuate epileptiform activity in the CA_3_ region of the immature rat hippocampus. *Epilepsy Research*.

[110] Dalby N. O. (2003). Inhibition of *γ*-aminobutyric acid uptake: anatomy, physiology and effects against epileptic seizures. *European Journal of Pharmacology*.

[111] Dalby N. O., Thomsen C., Fink-Jensen A. (1997). Anticonvulsant properties of two GABA uptake inhibitors NNC 05-2045 and NNC 05-2090, not acting preferentially on GAT-1. *Epilepsy Research*.

[112] Kersanté F., Rowley S. C. S., Pavlov I. (2013). A functional role for both *γ*-aminobutyric acid (GABA) transporter-1 and GABA transporter-3 in the modulation of extracellular GABA and GABAergic tonic conductances in the rat hippocampus. *Journal of Physiology*.

[113] Yoon B.-E., Lee C. J. (2014). GABA as a rising gliotransmitter. *Frontiers in Neural Circuits*.

[114] Ben-Ari Y. (2002). Excitatory actions of GABA during development: the nature of the nurture. *Nature Reviews Neuroscience*.

[115] Kahle K. T., Deeb T. Z., Puskarjov M. (2013). Modulation of neuronal activity by phosphorylation of the K-Cl cotransporter KCC2. *Trends in Neurosciences*.

[116] Kahle K. T., Staley K. J., Nahed B. V. (2008). Roles of the cation-chloride cotransporters in neurological disease. *Nature Clinical Practice Neurology*.

[117] Kaila K., Ruusuvuori E., Seja P., Voipio J., Puskarjov M. (2014). GABA actions and ionic plasticity in epilepsy. *Current Opinion in Neurobiology*.

[118] Puskarjov M., Ahmad F., Kaila K., Blaesse P. (2012). Activity-dependent cleavage of the K-Cl cotransporter KCC2 mediated by calcium-activated protease calpain. *The Journal of Neuroscience*.

[119] Bowery N. G. (1997). Metabotropic GABA(B) receptors cloned at last. *Trends in Pharmacological Sciences*.

[120] Gaiarsa J.-L., Kuczewski N., Porcher C. (2011). Contribution of metabotropic GABA_B_ receptors to neuronal network construction. *Pharmacology & Therapeutics*.

[121] Chalifoux J. R., Carter A. G. (2011). GABAB receptor modulation of synaptic function. *Current Opinion in Neurobiology*.

[122] Gähwiler B. H., Brown D. A. (1985). GABAB-receptor-activated K+ current in voltage-clamped CA3 pyramidal cells in hippocampal cultures. *Proceedings of the National Academy of Sciences of the United States of America*.

[123] Scholz K. P., Miller R. J. (1991). GABA_B_ receptor-mediated inhibition of Ca^2+^ currents and synaptic transmission in cultured rat hippocampal neurons. *The Journal of Physiology*.

[124] Mintz I. M., Bean B. P. (1993). GABA_B_ receptor inhibition of P-type Ca^2+^ channels in central neurons. *Neuron*.

[125] Mann E. O., Kohl M. M., Paulsen O. (2009). Distinct roles of GABA_A_ and GABA_B_ receptors in balancing and terminating persistent cortical activity. *Journal of Neuroscience*.

[126] Vergnes M., Boehrer A., Simler S., Bernasconi R., Marescaux C. (1997). Opposite effects of GABA_B_ receptor antagonists on absences and convulsive seizures. *European Journal of Pharmacology*.

[127] Prosser H. M., Gill C. H., Hirst W. D. (2001). Epileptogenesis and enhanced prepulse inhibition in GABA_B1_-deficient mice. *Molecular and Cellular Neuroscience*.

[128] Schuler V., Lüscher C., Blanchet C. (2001). Epilepsy, hyperalgesia, impaired memory, and loss of pre- and postsynaptic GABA_B_ responses in mice lacking GABA_B(1)_. *Neuron*.

[129] Kofler M., Kronenberg M. F., Rifici C., Saltuari L., Bauer G. (1994). Epileptic seizures associated with intrathecal baclofen application. *Neurology*.

[130] Princivalle A. P., Duncan J. S., Thom M., Bowery N. G. (2003). GABA_B1a_, GABA_B1b_ AND GABA_B2_ mRNA variants expression in hippocampus resected from patients with temporal lobe epilepsy. *Neuroscience*.

[131] Dugladze T., Maziashvili N., Börgers C. (2013). GABA_B_ autoreceptor-mediated cell type-specific reduction of inhibition in epileptic mice. *Proceedings of the National Academy of Sciences of the United States of America*.

[132] Houser C. R., Miyashiro J. E., Swartz B. E., Walsh G. O., Rieh J. R., Delgado-Escueta A. V. (1990). Altered patterns of dynorphin immunoreactivity suggest mossy fiber reorganization in human hippocampal epilepsy. *Journal of Neuroscience*.

[133] Perez Y. (1996). Axonal sprouting of CA1 pyramidal cells in hyperexcitable hippocampal slices of kainate-treated rats. *European Journal of Neuroscience*.

[134] Zhang W., Yamawaki R., Wen X. (2009). Surviving hilar somatostatin interneurons enlarge, sprout axons, and form new synapses with granule cells in a mouse model of temporal lobe epilepsy. *Journal of Neuroscience*.

[135] Peng Z., Zhang N., Wei W. (2013). A reorganized GABAergic circuit in a model of epilepsy: evidence from optogenetic labeling and stimulation of somatostatin interneurons. *Journal of Neuroscience*.

[136] Liu Y.-Q., Yu F., Liu W.-H., He X.-H., Peng B.-W. (2014). Dysfunction of hippocampal interneurons in epilepsy. *Neuroscience Bulletin*.

[137] Sun C., Sun J., Erisir A., Kapur J. (2014). Loss of cholecystokinin-containing terminals in temporal lobe epilepsy. *Neurobiology of Disease*.

[138] Araque A., Carmignoto G., Haydon P. G. (2001). Dynamic signaling between astrocytes and neurons. *Annual Review of Physiology*.

[139] Shelton M. K., McCarthy K. D. (1999). Mature hippocampal astrocytes exhibit functional metabotropic and ionotropic glutamate receptors in situ. *Glia*.

[140] Porter J. T., McCarthy K. D. (1996). Hippocampal astrocytes in situ respond to glutamate released from synaptic terminals. *Journal of Neuroscience*.

[141] Araque A., Martín E. D., Perea G., Arellano J. I., Buño W. (2002). Synaptically released acetylcholine evokes Ca^2+^ elevations in astrocytes in hippocampal slices. *Journal of Neuroscience*.

[142] Eid T., Hammer J., Rundén-Pran E. (2007). Increased expression of phosphate-activated glutaminase in hippocampal neurons in human mesial temporal lobe epilepsy. *Acta Neuropathologica*.

[143] Wetherington J., Serrano G., Dingledine R. (2008). Astrocytes in the epileptic brain. *Neuron*.

[144] Gómez-Gonzalo M., Losi G., Chiavegato A. (2010). An excitatory loop with astrocytes contributes to drive neurons to seizure threshold. *PLoS Biology*.

[145] Pascual O., Ben Achour S., Rostaing P., Triller A., Bessis A. (2012). Microglia activation triggers astrocyte-mediated modulation of excitatory neurotransmission. *Proceedings of the National Academy of Sciences of the United States of America*.

[146] Tang F.-R., Lee W.-L. (2001). Expression of the group II and III metabotropic glutamate receptors in the hippocampus of patients with mesial temporal lobe epilepsy. *Journal of Neurocytology*.

[147] de Lanerolle N. C., Eid T., von Campe G., Kovacs I., Spencer D. D., Brines M. (1998). Glutamate receptor subunits GluR1 and GluR2/3 distribution shows reorganization in the human epileptogenic hippocampus. *European Journal of Neuroscience*.

[148] Steinhäuser C., Seifert G. (2002). Glial membrane channels and receptors in epilepsy: impact for generation and spread of seizure activity. *European Journal of Pharmacology*.

[149] Nagaraja R. Y., Grecksch G., Reymann K. G., Schroeder H., Becker A. (2004). Group I metabotropic glutamate receptors interfere in different ways with pentylenetetrazole seizures, kindling, and kindling-related learning deficits. *Naunyn-Schmiedeberg’s Archives of Pharmacology*.

[150] Nagaraja R. Y., Becker A., Reymann K. G., Balschun D. (2005). Repeated administration of group I mGluR antagonists prevents seizure-induced long-term aberrations in hippocampal synaptic plasticity. *Neuropharmacology*.

[151] Carmignoto G., Fellin T. (2006). Glutamate release from astrocytes as a non-synaptic mechanism for neuronal synchronization in the hippocampus. *Journal of Physiology Paris*.

[152] Tian G.-F., Azmi H., Takano T. (2005). An astrocytic basis of epilepsy. *Nature Medicine*.

[153] Fellin T., Gomez-Gonzalo M., Gobbo S., Carmignoto G., Haydon P. G. (2006). Astrocytic glutamate is not necessary for the generation of epileptiform neuronal activity in hippocampal slices. *Journal of Neuroscience*.

[154] Angulo M. C., Kozlov A. S., Charpak S., Audinat E. (2004). Glutamate released from glial cells synchronizes neuronal activity in the hippocampus. *Journal of Neuroscience*.

[155] Crunelli V., Carmignoto G. (2013). New vistas on astroglia in convulsive and non-convulsive epilepsy highlight novel astrocytic targets for treatment. *Journal of Physiology*.

[156] Navarrete M., Araque A. (2008). Endocannabinoids mediate neuron-astrocyte communication. *Neuron*.

[157] Hofmann M. E., Frazier C. J. (2013). Marijuana, endocannabinoids, and epilepsy: potential and challenges for improved therapeutic intervention. *Experimental Neurology*.

[158] Coiret G., Ster J., Grewe B. (2012). Neuron to astrocyte communication via cannabinoid receptors is necessary for sustained epileptiform activity in rat hippocampus. *PLoS ONE*.

[159] Mariotti L., Losi G., Sessolo M., Marcon I., Carmignoto G. (2016). The inhibitory neurotransmitter GABA evokes long-lasting Ca^2+^ oscillations in cortical astrocytes. *Glia*.

[160] Kang J., Jiang L., Goldman S. A., Nedergaard M. (1998). Astrocyte-mediated potentiation of inhibitory synaptic transmission. *Nature Neuroscience*.

[161] Liu Q., Xu Q., Kang J., Nedergaard M. (2004). Astrocyte activation of presynaptic metabotropic glutamate receptors modulates hippocampal inhibitory synaptic transmission. *Neuron Glia Biology*.

[162] Semyanov A., Kullmann D. M. (2000). Modulation of GABAergic signaling among interneurons by metabotropic glutamate receptors. *Neuron*.

[163] Somogyi P., Dalezios Y., Luján R., Roberts J. D. B., Watanabe M., Shigemoto R. (2003). High level of mGluR7 in the presynaptic active zones of select populations of GABAergic terminals innervating interneurons in the rat hippocampus. *European Journal of Neuroscience*.

[164] Kogo N., Dalezios Y., Capogna M., Ferraguti F., Shigemoto R., Somogyi P. (2004). Depression of GABAergic input to identified hippocampal neurons by group III metabotropic glutamate receptors in the rat. *European Journal of Neuroscience*.

[165] Jouvenceau A., Dutar P., Billard J.-M. (1995). Presynaptic depression of inhibitory postsynaptic potentials by metabotropic glutamate receptors in rat hippocampal CA1 pyramidal cells. *European Journal of Pharmacology*.

[166] Lalo U., Palygin O., Rasooli-Nejad S., Andrew J., Haydon P. G., Pankratov Y. (2014). Exocytosis of ATP from astrocytes modulates phasic and tonic inhibition in the neocortex. *PLoS Biology*.

